# Malaria Epidemics and Surveillance Systems in Canada

**DOI:** 10.3201/eid1007.030826

**Published:** 2004-07

**Authors:** J. Dick MacLean, Anne-Marie Demers, Momar Ndao, Evelyne Kokoskin, Brian J. Ward, Theresa W. Gyorkos

**Affiliations:** * Montreal General Hospital, McGill University Centre for Tropical Diseases, Montreal, Québec, Canada;; † McGill University, Montreal, Québec, Canada

**Keywords:** malaria, surveillance, epidemic, Canada, perspective

## Abstract

In the past decade, fluctuations in numbers of imported malaria cases have been seen in Canada. In 1997 to 1998, malaria case numbers more than doubled before returning to normal. This increase was not seen in any other industrialized country. The Canadian federal malaria surveillance system collects insufficient data to interpret these fluctuations. Using local (sentinel), provincial, federal, and international malaria surveillance data, we evaluate and interpret these fluctuations. Several epidemics are described. With an ever-increasing immigrant and refugee population of tropical origin, improved surveillance will be necessary to guide public health prevention policy and practice. The Canadian experience is likely to be generalizable to other industrialized countries where malaria is a reportable disease within a passive surveillance system.

## Abstract 2

Malaria surveillance data are evaluated for causes of epidemics in Canada.

Malaria has been a reportable communicable disease in Canada since 1929, when a surveillance system for communicable diseases was first developed. Although no longer endemic in Canada, malaria has remained an important imported disease, principally in immigrants and travelers (1–3). Rarely, it has been transmitted in blood products (4). Published reports document delays in clinical and laboratory diagnoses of malaria and lack of understanding of malaria prophylaxis and fever management in travelers (3). The Canadian infectious disease surveillance system has reported an average of 538 malaria cases per year since 1990, and Statistics Canada reported an average of one death per year (5,6, Carole Scott [Division of Disease Surveillance, Health Canada], pers. comm.). The present federal surveillance system reports the age and sex of a patient and does not document malaria death rate, malaria species, nor the likely country of acquisition. While malaria-related deaths may be few, that any exist is a matter of concern. The continued incidence of malaria cases and deaths in Canada suggests that the malaria surveillance system should be strengthened and used more proactively to help identify appropriate preventive measures.

All 10 provincial and 3 territorial health authorities in Canada are required by law to report diagnoses of malaria and other selected diseases to federal authorities at Health Canada (2). Summary reports of these diseases are published by both levels of reporting in provincial and territorial news bulletins and by Health Canada in the Canada Communicable Diseases Report.

In several instances over the past decade, malaria incidence in Canada as a whole, or in individual provinces, reached epidemic levels (7). Why some were not immediately identified and why no comprehensive analysis has been published as part of government surveillance systems are questions that will be addressed. Failing to recognize these epidemics has limited the ability of public health officials to assess and intervene appropriately to control the illness and death associated with imported malaria in Canada.

This study evaluated and summarized data collected over the past 22 years by local, provincial, and federal malaria surveillance systems, from Canadian federal immigration and refugee data resources and from international tourist resources, to identify and explore the causes of malaria epidemics. In addition, geographic patterns and *Plasmodium* spp. profiles of malaria are examined. This analysis led us to conclude that changes are needed in both the surveillance reporting instruments and how these surveillance results are analyzed and used.

## Methods

The databases used for the present analysis include 22 years of records from a local malaria reference center in Montreal, Canada (the McGill University Centre for Tropical Diseases [TDC]) and up to 13 years of quality assurance and notifiable disease surveillance databases of the provincial and federal governments of Canada, France, India, Switzerland, the United States, and the United Kingdom. TDC is a clinical and laboratory facility that provides care to 800 to 1,100 new patients per year (approximately 60% primary cases and 40% consult cases), drawn mainly from the Montreal region. The TDC database from 1981 to 2002 has allowed previous detailed reviews of changing patterns of malaria in its patient populations (8-10). Malaria-relevant data captured include category of traveler (tourist, immigrant, refugee, expatriate, missionary, and volunteer), countries visited, and malaria species. A diagnosis of malaria is made if parasites are noted on a blood smear (thin, thick, or buffy coat) or if, in the last 5 years, the patient had a positive result on a malaria antigen-capture test (e.g., Macromed [Nova Century Scientific, Inc., Burlington, Ontario, Canada], ICT Malaria P.f. [ICT Diagnostics, Brookvale, New South Wales, Australia], or OptiMAL [Flow Inc., Portland, OR]). While active surveillance studies during this period included polymerase chain reaction (PCR) as a screening tool, PCR-positive cases were not included in any of the passive surveillance statistics unless they were also independently confirmed by either malaria antigen capture or smear.

Provincial reportable disease databases have included, in the past 10 years, patient characteristics such as age, sex, and malaria species, but not the likely country of acquisition. Because 90% of all malaria cases in Canada were reported by the Provinces of British Columbia (Monica Naus [British Columbia Centre for Disease Control], pers. comm.), Ontario (Lorraine Schiedel [Ontario Ministry of Health and Long-Term Care], pers. comm.), and Québec (Colette Colin [Ministère de la santé et des services sociaux, Québec], pers. comm.), the present analysis focuses on their data, primarily for 1990-2002 (11). Quality assurance data for the province of Québec (1994-2002) were provided by TDC and the Laboratoire de Santé Publique du Québec. The federal government's notifiable disease database from 1990 to 2002 is a compilation of selected information from individual provincial databases and includes patient age and sex for each report but no malaria species or country of acquisition (Carole Scott [Division of Disease Surveillance, Health Canada], pers. comm.).

International malaria surveillance data (1990-2002) were acquired from the World Health Organization (WHO) Regional Office for South East Asia (Rakish Mani Rastogi, pers. comm.), the WHO Regional Office for Europe (12), and the United States (13-24). Malaria rates for all countries were based on population data of the U.S. Census Bureau (25).

Trends in Canadian immigration and refugee data for the years 1990-2002 were provided by Citizen and Immigration, Canada (Karen Tremblett [Medical Services Branch, Citizen and Immigration Canada], pers. comm.), data on language by Statistics Canada (26), and travel patterns of Canadians to the tropics by the World Tourism Organization, Madrid (27).

## Results

### TDC Database

**Figure 1:**
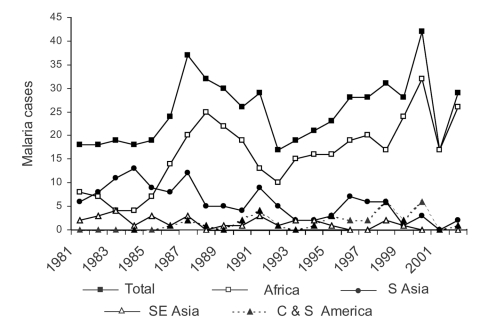
McGill University Centre for Tropical Diseases malaria cases by year and origin (N = 553).

**Figure 2:**
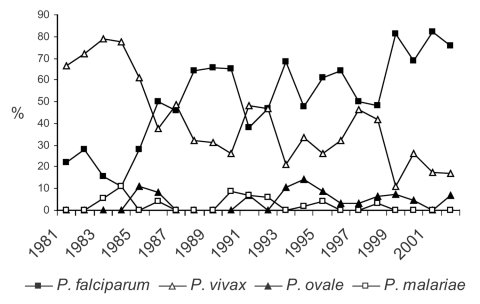
Relative rates of *Plasmodium* species (N = 553).

Overall, 553 clinical cases of malaria were seen at TDC from 1981 to 2002, with some fluctuation over time but an overall gradual increase (Figure 1). In these 553 cases, 562 microscopy diagnoses were made; *Plasmodium falciparum* 295 (52%), *P. vivax* 218 (39%), *P. ovale* 26 (5%), *P. malariae* 16 (3%), and unknown species 7 (1%). Nine (2%) of the clinical cases were mixed infections, involving *P. falciparum* with either *P. malariae* or *P. vivax.* Seven patients were seen two or three times with relapses of *P. vivax* (recurrence >2 months later). The relative frequency of species changed over time, with a gradual increase in the proportion of *P. falciparum* cases from 20% to 30% in the early 1980s to 60% to 70% in the 1990s and to 70% to 80% in the present decade (Figure 2). Over this 22-year period, only one fatality occurred (3).

Sixty-one countries were identified as the most likely sources of the malaria exposure. Sub-Saharan Africa was the region where most patients contracted malaria, 353 case-patients (65%), followed by south Asia (23%), Southeast Asia (6%), Central America (5%), and South America (1%). However, India, with 110 cases (20%), was the single most frequent source country. Tourists (29%), immigrants or refugees (29%), and foreign workers (24%) represented the categories most frequently reported. A shift over time occurred in the importance of sub-Saharan Africa as a source of malaria cases. In the 1980s, 50% of malaria infections were acquired in Africa; in the 90s, 70%; and, since 2000, 85%. Patients of all categories were more likely to contract *P. falciparum* in Africa (74.3%) while it has been an uncommon species in south Asia (5.8%). The increase in *P. falciparum* cases over time correlated with the increase in the total number of malaria cases contracted in Africa; *P. falciparum* represented ≈30% of all cases in the early 1980s and increased to 70% in the late 1990s. From 1981 to 2002, 96% of malaria infections acquired in south Asia were non-falciparum malarias, while only 29% of infections from Africa were non-falciparum. None of the 553 cases of malaria originated from China, Malaysia, Saudi Arabia, Peru, or Venezuela, which are frequent travel destinations of Québecers. Other common travel destinations contributed little to the 20-year malaria case total (e.g., Philippines [1 case], South Africa [1], Costa Rica [2], Mexico [2], and Dominican Republic [3]). Malaria cases from Africa from 1992 to 2000 came predominantly (69%) from the French-speaking African countries, notably higher than the proportion of U.S. travelers (18%) who acquired malaria in these countries (13–24).

Two "epidemics" were observed at TDC during this period (Figures 1 and 2). The first was in 1986 to 1990 and resulted from increasing numbers of *P. falciparum* infections from Africa, thought to be due to increasing chloroquine-resistant *P. falciparum* in chloroquine-prophylaxed travelers (28), and the second was in 1999 through 2000, resulting from increased numbers of *P. falciparum* infections associated with the arrival in Québec of large numbers of refugees from Tanzanian refugee camps (29-30). Both epidemics were recognized and reported in the literature soon after their appearance.

### Federal and Provincial Databases

A review of the Federal Health Canada databases for the incidence of malaria in Canada, from 1990 through 2002, documents a range from 364 to 1,029 cases per year, with an average of 538 cases per year during the period (or an average of ≈1.8 cases per 100,000 population per year) (6) (Carole Scott [Division of Disease Surveillance, Health Canada], pers. comm.).

**Figure 3:**
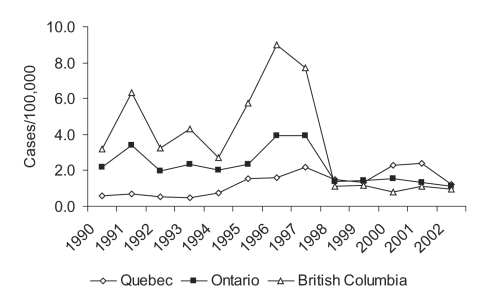
Provincial malaria rates for Québec, Ontario, and British Columbia (6,11, Colette Colin [Ministère de la santé et des services sociaux, Québec], pers. comm.; Lorraine Schiedel [Ontario Ministry of Health and Long Term Care], pers. comm.; Monica Naus [British Columbia Centre for Disease Control], pers. comm.; Carole Scott [Division of Disease Surveillance, Health Canada], pers. comm.).

**Figure 4:**
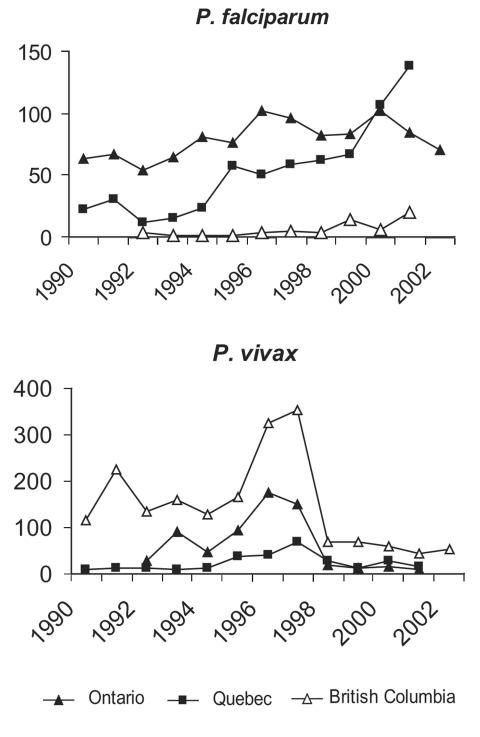
*Plasmodium* species provincial trends (6,11, Colette Colin [Ministère de la santé et des services sociaux, Québec], pers. comm.; Monica Naus [British Columbia Centre for Disease Control], pers. comm.; Lorraine Schiedel [Ontario Ministry of Health and Long Term Care], pers. comm.).

**Figure 5:**
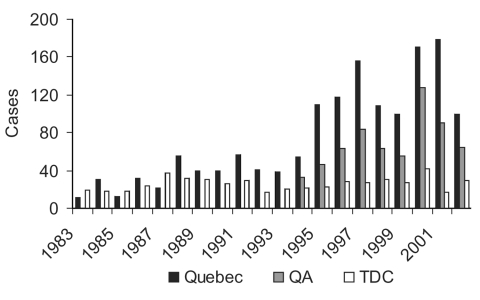
Malaria surveillance of Québec province, McGill Centre for Tropical Diseases (TDC), and Québec quality assurance (QA) program (Colette Colin [Ministère de la santé et des services sociaux, Québec], pers. comm.).

While all ages were affected, patients were mostly adults from 20 to 59 years of age. A similar pattern of malaria incidence was observed in males and females. British Columbia had the highest rate per 100,000 (3.6 ±2.8) over this period, followed by Ontario (2.2 ± 0.98), and Québec (1.3 ± 0.67) (Figure 3). However, the highest cumulative numbers for the 12-year period were reported from Ontario (N = 3,222), followed by British Columbia (N = 1,763), and Québec (N = 1,246). The Canadian data suggest that an epidemic occurred from 1995 to 1997, reflecting higher than average numbers of malaria cases in these years from British Columbia, Ontario, and to a lesser extent, from Québec (Figure 3). This epidemic was almost entirely due to increased P. vivax being reported in these provinces (Figure 4). From 1990 to 1999, two events occurred in Québec that did not occur in other Canadian provinces. In 1994, a quality assurance program for the province was initiated by TDC, in collaboration with the Laboratoire de Santé Publique du Québec. This three-pronged program provided: 1) a free, rapid turnaround confirmation service for positive or equivocal malaria diagnoses from any laboratory in Québec, 2) a biannual malaria-training course for clinical laboratory technologists, and 3) a voluntary proficiency testing program for Québec hospital laboratories, in which once or twice a year they are sent unknown positive and negative smears for identification and receive extensive feedback. From the inception of the quality assurance program, a parallel increase was seen in numbers of specimens being sent to the reference laboratory and to the Québec surveillance program (Figure 5). This fourfold increase represented an epidemic attributable to improved diagnosis and reporting. The second event in Québec was another epidemic, in this case of falciparum malaria, observed in 2000 to 2001 and associated with a large influx of refugees from Tanzanian refugee camps (Figure 4) (29).

### International Malaria Surveillance

**Figure 6:**
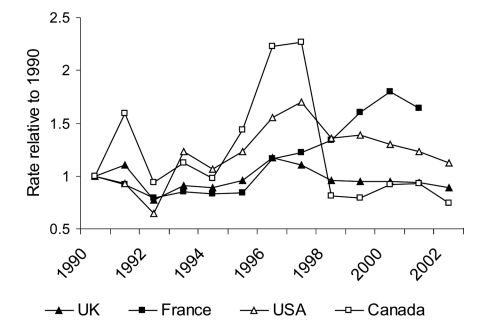
Malaria cases/100,000 relative to 1990 (6,12–25,30, Carole Scott [Division of Disease Surveillance, Health Canada], pers. comm.).

**Figure 7:**
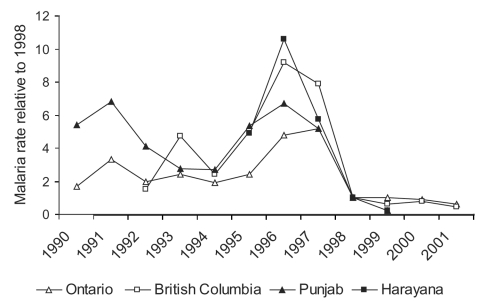
*Plasmodium vivax* incidence relative to 1998 (6,11, Colette Colin [Ministère de la santé et des services sociaux, Québec], pers comm.; Monica Naus [British Columbia Centre for Disease Control], pers. comm.).

National surveillance systems for malaria are far from universal, and compliance with national surveillance instruments, when measured, is low. The stability of the degree of underreporting over time has been not been evaluated. Despite these limitations, trends in malaria incidence over time in different countries can provide useful information. From 1995 to 1997, when parts of Canada were having malaria epidemics, similar but smaller changes in malaria rates were observed in the United States and United Kingdom (Figure 6). An examination of the geographic origin of malaria cases reported in the United States in the mid-1990s showed a more than twofold increase in malaria cases imported from India in 1995 through 1997, with an abrupt drop in these cases in 1998 (12-23). During this same period, a similar epidemic of *P. vivax* malaria occurred in certain states in India known to have important immigration and travel links with North America (Figure 7). During the 1990s, France had a 60% increase in malaria in the latter part of the decade (31), reportedly caused by African travel, and Denmark experienced an increase of 68%; Germany, Italy, Spain, Sweden, the Netherlands, and Belgium, however, had stable rates during this time (12,31). None of these countries increase in malaria seen Canada and, to a lesser degree, United States from 1995 to 1997.

## Discussion

Malaria importations into Canada can occur by either immigration or travel, and changing malaria attack rates in the countries of exposure are likely to influence the incidence of imported disease. Changes in Canadian immigration and refugee patterns from 1990 to 2002 are notable for a threefold increase in annual immigrant numbers from the Indian subcontinent and relatively stable numbers from sub-Saharan Africa. Neither combined nor separate provincial immigration and refugee patterns explain the important swings in annual Canadian malaria rates.

While the geographic origins of immigrants and refugees do not immediately explain the epidemic changes in *P. vivax* malaria seen in the mid-1990s, their nonrandom aggregation in certain provinces allows additional insights. African immigrants and refugees have settled all across Canada in every province in numbers that paralleled the province's population. Immigrants and refugees from the Indian subcontinent did not: 84% settled in Ontario and British Columbia, the provinces with the most pronounced *P. vivax* epidemics. Canadian travelers to malaria-endemic areas have gradually but steadily increased during the past 15 years, most notably with a threefold increase to Southeast Asia and Central and South America, a twofold increase to the important malarial region of south Asia, and a smaller increase to Africa. Travel patterns did not offer an explanation for either the *P. vivax* epidemics in British Columbia and Ontario in the late 1990s or the *P. falciparum* epidemic in Québec from 2000 to 2001. The World Tourism Organization data do not break down Canadian travel by traveler's province of origin; however, comparing U.S. malaria surveillance data with TDC surveillance data, both of which track the likely country of origin of a malaria case, Québec travelers acquire most African malaria in French-speaking African countries (69%), a minor source of malaria for Americans (18%). English-speaking Ontario and British Columbia likely have more "American" travel patterns than francophone Québecers. However, no fluctuations were seen in rates of travel to either East or West Africa or to the Indian subcontinent, the major source of Canada-acquired *P. vivax* malaria, which would explain the impressive change in Canadian malaria reporting from 1995 through 1997.

The two surveillance sources of India and the United States were also reviewed for malaria incidence trends. American malaria surveillance includes the likely country of origin of a malaria case. An obvious increase in *P. vivax* cases from India was seen in the United States, from 150 cases to 371 and down to 123, during 1995 to 1997. This increase paralleled the epidemic peak seen in Canada, primarily in Ontario and British Columbia. In India, an epidemic of *P. vivax* malaria occurred during this same period (1995–1997) in the Punjabi states of Punjab and Haryana (Figure 7). With negligible changes in travel destination or immigration numbers to explain the 1995–1997 epidemic in Canada, the explanation is probably an increased *P. vivax* attack rate in Canadians traveling to the Punjab, where a *P. vivax* epidemic occurred and ended at the same time as the Canadian epidemic.

Canadian notifiable diseases surveillance data generated by local, provincial, and federal sources provided evidence for the occurrence of two as-yet unreported malaria epidemics in Canada in the last decade. One was a *P. vivax* epidemic, the epicenter of which was almost certainly in the Punjab, India. The second was a *P. falciparum* epidemic in Québec related to an increased influx of Central African refugees from Tanzanian refugee camps. At the time, neither of these epidemics was brought to the attention of health practitioners in travel clinics through publication or other standard channels. Consequently, possible explanations and potential interventions were not discussed.

Trends in immigration do not explain the malaria incidence changes seen in Canada. These trends differ for each province both in terms of country of origin and numbers. However, the major fluctuations in federal and provincial malaria rates from 1990 to 2002, and, in particular, during the epidemic years, were not found to be directly linked to provincial immigration numbers or to the travel destinations of Canadians in general. Unfortunately, no mechanism records the destinations of travelers from specific provinces. Ontario and British Columbia are home to 86% of the Punjabi-speaking Canadian population. If provincial travel destination data were available, it would likely show that these provinces were the source of most Canadian travelers to the Indian Punjab (27).

Working back from individual case data in each province seems to be the most accurate way to identify countries where large numbers of imported malaria may originate. Country of likely origin of the malaria should be indicated on all requisitions for malaria laboratory diagnosis, and this information and the malaria species should be reported to provincial and then federal surveillance bodies. The fact that the 1995–1997 epidemic was primarily due to *P. vivax*, the predominant malaria species in India, and that it occurred at the same time as the *P. vivax* epidemic in the Indian Punjabi states of Punjab and Haryana, is strong evidence to conclude that the Canadian epidemic was an extension of the Punjab epidemic. This association is supported by the abrupt halt of both Canadian and Punjabi epidemics in the same year.

The surveillance process for notifiable diseases in Canada and in other countries where malaria is now an imported disease should be reviewed. Specific conditions, such as the frequency of analysis of surveillance data, need to be discussed and agreed on by collectors of these data at each level of government. Without a firm plan in place for analysis and dissemination of results, the validity, not to mention the utility of the entire surveillance system, is placed in jeopardy. One approach could be the American emerging infections programs, a link between public health, academic, and clinical communities (32).

For surveillance data to be useful and cost-effective, it must be both available in a timely fashion and interpretable. Local surveillance systems have obvious benefits when increased water- and foodborne infections or vaccine-preventable diseases lead to quick public health action. Malaria surveillance differs in two major ways from these classical scenarios. Malaria is an imported disease, and no immediate intervention (e.g., vaccine, chemical disinfectant, and handwashing) will affect an epidemic. As with sexually transmitted infections, the control of a malaria epidemic in Canadian travelers requires public education. In the United States, both malaria speciation and country of likely acquisition of the malaria case are part of surveillance. Such information, if part of the Canadian system, would allow rapid appreciation of the etiology of epidemics such as those reviewed here, which would potentially lead to appropriate public health response.

Dr. MacLean is professor of medicine and director of the McGill University Centre for Tropical Diseases. His research interests are parasitic disease outbreak investigation (trichinosis, *Metorchis* infections, and malaria) and the development of diagnostic tests for the clinical parasitology laboratory.
